# Relapse of skull osteoma after hydroxyapatite cement cranioplasty: Case Report

**DOI:** 10.3389/fonc.2023.1174128

**Published:** 2023-05-19

**Authors:** Xiang Yang, Yanhui Liu, Yuekang Zhang

**Affiliations:** Department of Neurosurgery, West China Hospital of Sichuan University, Chengdu, Sichuan, China

**Keywords:** hydroxyapatite cement (HAC), osteoma, cranioplasty, dura, pathogenesis

## Abstract

In this case report, we present an extremely rare and previously unreported case of skull osteoma relapse without any attachment to the skull after hydroxyapatite cement (HAC) cranioplasty. The 49-year-old male patient was admitted with recurrence of the left frontal skull lesion; he underwent craniectomy and HAC cranioplasty for a left frontal osteoma 14 years before. Intraoperative findings disclosed multiple irregular lesions located on the HAC flap without any attachment to the bony structure and the roots of the lesions originating from the outer layer of the dura through several reserved holes. Pathological diagnosis was osteoma. The purpose of this report is to document this rare occurrence and provide the most probable pathogenesis for this rare event.

## 1 Introduction

Osteomas are benign neoplasms with mature normal osseous tissue. They often occur on the long bones of the extremities, in the sinuses of the facial bones, in the skull, and in the mandible (1). Hydroxyapatite cement (HAC) cranioplasty is widely used for repair of a variety of cranial defects; especially, it can adjust the shape and size of HAC flap to surgery conditions due to its biocompatibility and favorable tensile properties (2, 3). However, skull osteoma relapse without any attachment to the skull after HAC cranioplasty is extremely rare, and no case has been reported so far. Here, we report one case of multiple osteomas relapse after HAC cranioplasty, without any connections with the skull, which is firstly reported in the literature to our knowledge.

## 2 Case presentation

### 2.1 Presentation and examination

A 49-year-old male patient was admitted with recurrence of the left frontal skull mass; he underwent craniectomy and HAC cranioplasty for a left frontal skull osteoma 14 years before. Unfortunately, the clinical data from his first visit have been lost at the local hospital, and neither the patient nor his family know what material was used to repair the skull defect on the first surgery. Subsequently, three small hard masses could be touched under the scalp within the surgical area on the third year after surgery, and the masses could not be moved. There was no local tenderness and scalp swelling. The patient chose conservative treatment because of no obvious discomfort. However, the masses slowly grew up and affected the appearance gradually in the following 10 years. The physical examination revealed three distinct immovable masses protruding from the skin surface of the left forehead. They were hard in texture and smooth in edge, closely related to the so-called skull. The other physical examination and laboratory test (including colonoscopy) results were normal.

**Figure 1:**
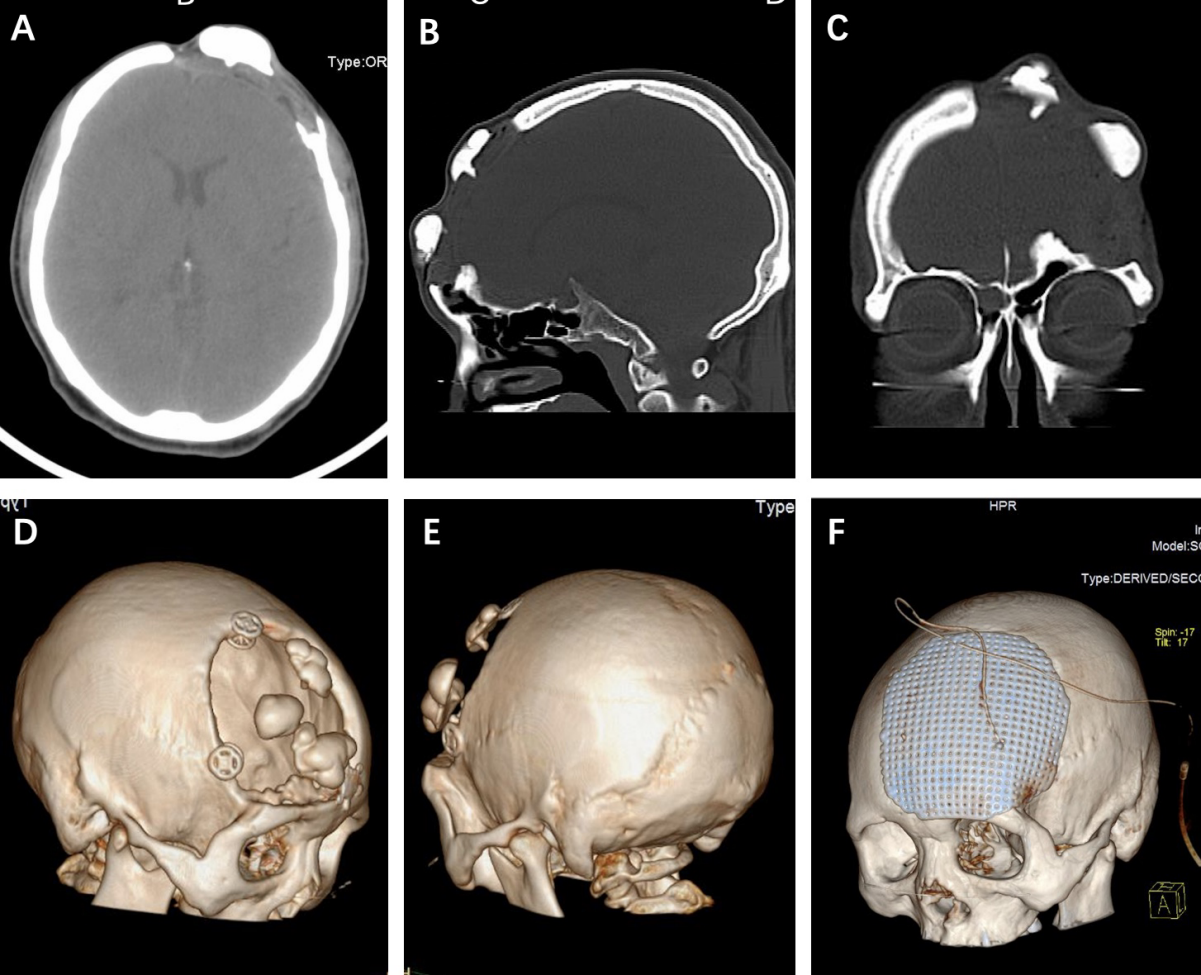
**(A-C)** Axial, sagittal, and coronary CT scans showed multiple hyperdense lesions without the involvement of bony structure. **(D, E)** 3D-CT skull reconstruction revealed the multiple lesions located within the cranial defects and internal fixations placed at the edge of the skull defect. **(F)** The postoperative 3D-CT skull reconstruction of the titanium mesh cranioplasty.

**Figure 2:**
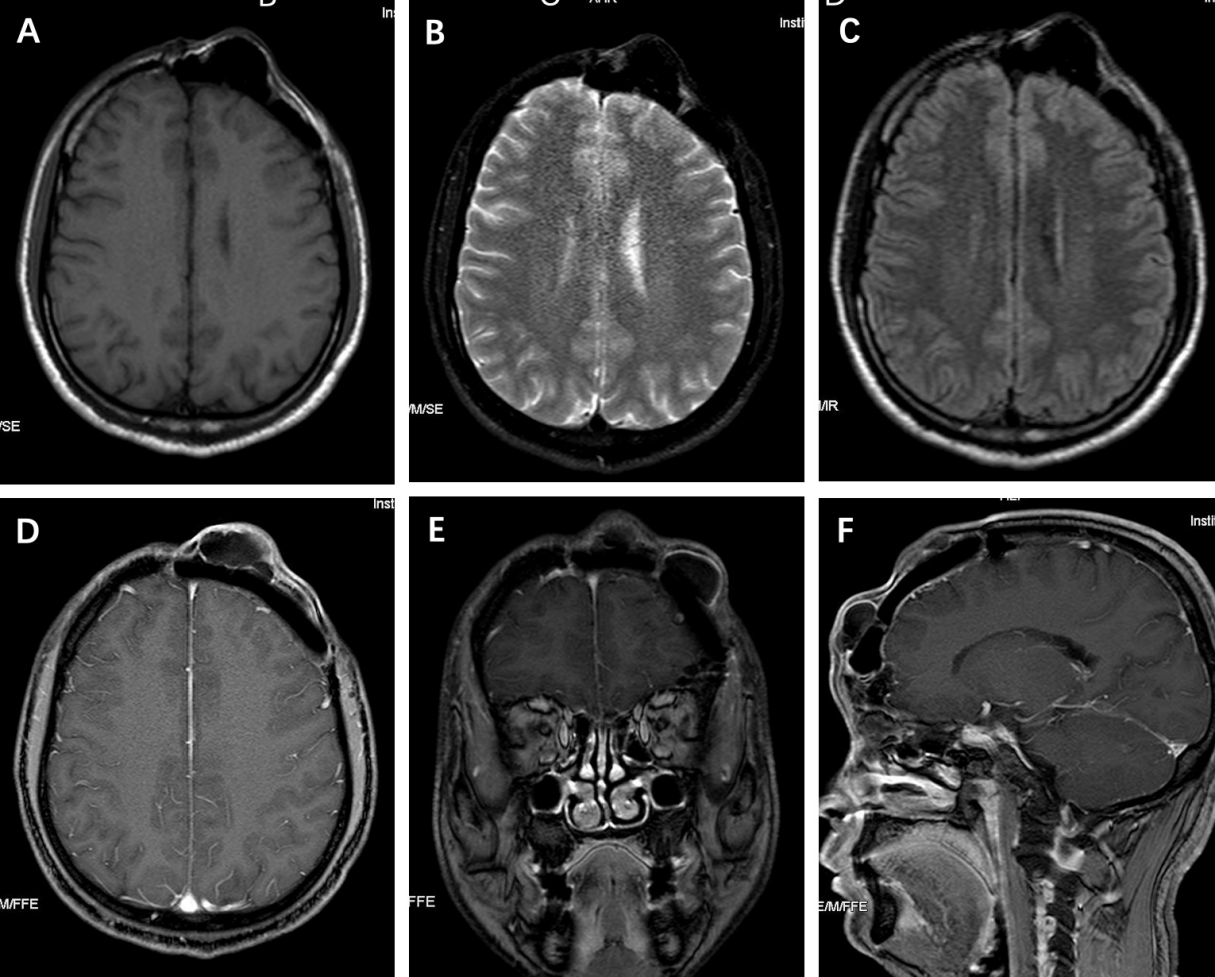
**(A-F)** All the MRI sequences depicted the lesions’ low signals.

CT scans showed partial defect of the left frontal skull with internal fixation shadow and multiple high-density nodules with a similar density as bone (**Figures 1A–C**). 3D-CT skull reconstruction revealed multiple lesions located within the left frontal cranial defects and three internal fixations placed at the edge of the cranial defects (**Figures 1D, E**). The MRI findings revealed multiple low-density signal lesions located in the left frontal cranial defect area without meningoencephalocele. All the MRI sequences showed low signals (**Figure 2**).

The preoperative diagnosis of the osteoma recurrence was made based on the limited information provided by the patient. The operation was performed with adequate preparation and a lot of doubts, especially for how to reasonably interpret the preoperative imaging findings.

### 2.2 Surgical treatment

**Figure 3:**
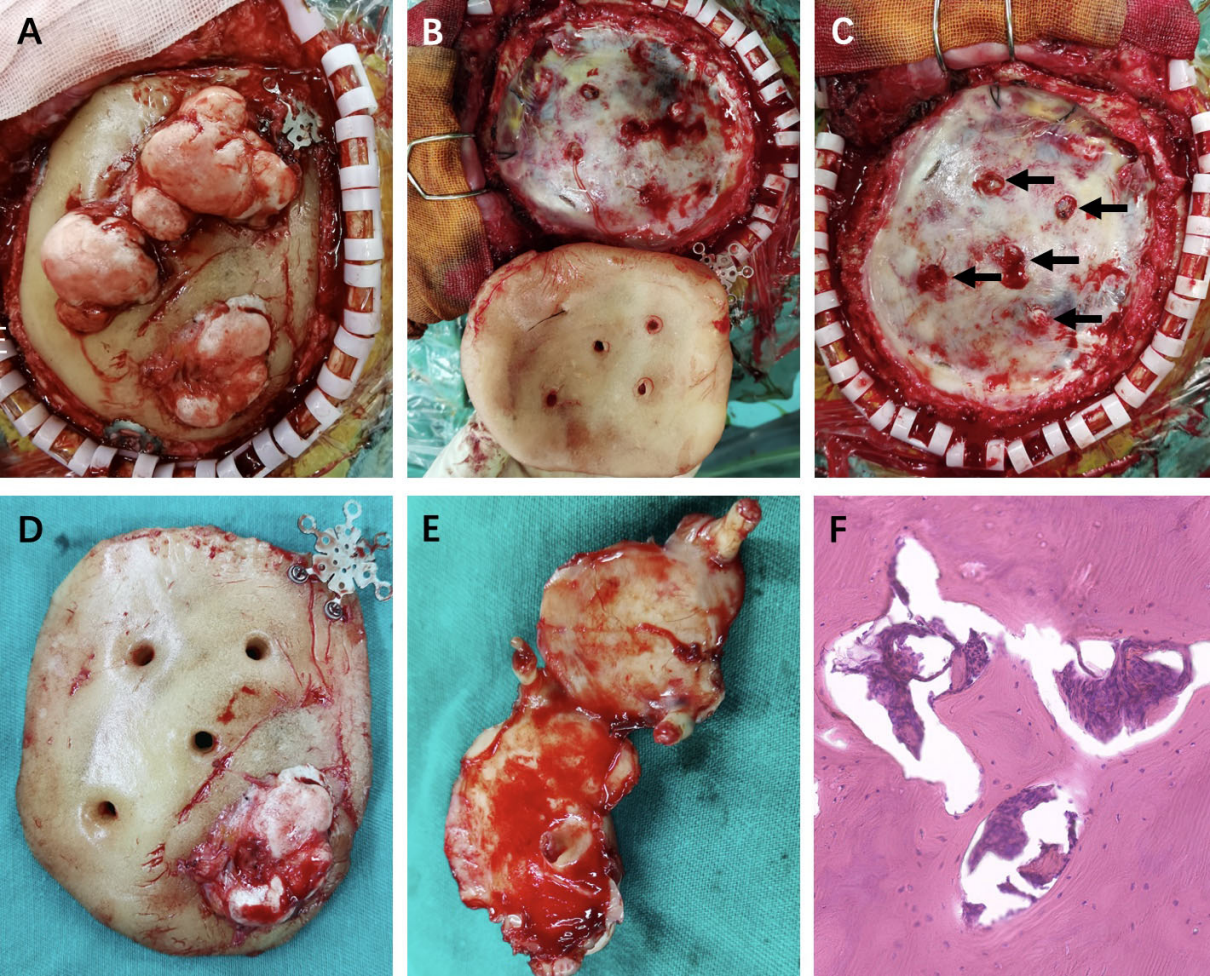
**(A)** The multiple irregular lesions were on the HAC flap with three internal fixations. **(B, C)** The lesions originated from the outer layer of the intact dura matter through the HAC holes (black arrows). **(D, E)** The HAC flap and the exposure of lesions in ventral area with roots. **(F)** Pathological diagnosis was osteoma hematoxylin and eosin (magnification, ×200).

The intraoperative findings disclosed that the multiple irregular lesions were nodular and hard and showed obvious ossification, and they were on the HAC flap (**Figures 3A, D**). The HAC was fixed by three internal fixations (**Figures 3A, B, D**). Intraoperatively, the lesions were found to originate from the surface of the dura mater and grew out through the reserved HAC holes, which were used to suspend the dura mater on the first surgery (**Figures 3B–D**). The lesions just adhered closely to the HAC surface without any attachment to the bony structure (**Figures 3A, D, E**). The dura matter was intact, and the roots of the lesions were given to cauterization (**Figure 3C**) (black arrows). The patient received titanium mesh cranioplasty (**Figure 1F**).

### 2.3 Postoperative diagnosis and follow-up

**Figure 4:**
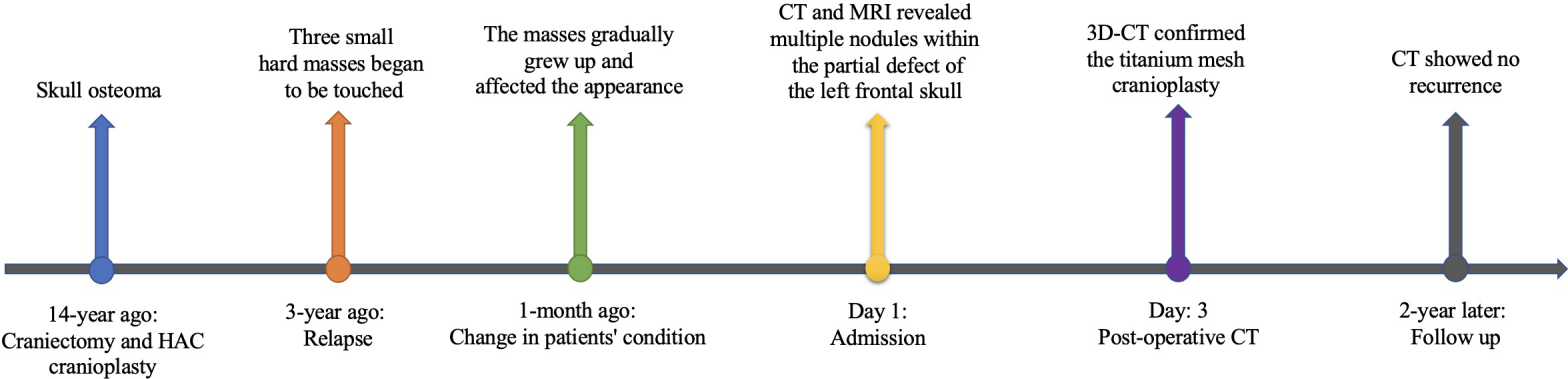
Timeline of the symptoms, management, and outcomes of the patient.

Pathology suggested the diagnosis of osteoma (**Figure 3F**). The tumors did not recur during the 2-year follow-up period according to the repeated cranial CT at the local hospital. The patient was administrated to long-term follow-up supervision considering the rare characteristics of his disease. The timeline of the symptoms, management, and outcomes of the patient are summarized in **Figure 4**.

## 3 Discussion

Osteomas are benign tumors consisting of mature normal osseous tissue, commonly encountered on long bones and in the mandible, sinuses of the facial bones, and the skull. The skull osteomas often have a good outcome without recurrence after surgical removal; however, they usually require cranioplasty (1, 4). Hydroxyapatite is a rapid-setting polymer formed by the mixture of calcium phosphate powder with a pH-neutral liquid (3). HAC is widely used for repair of a variety of cranial defects due to its biocompatibility and favorable tensile properties (2, 3). Adoption of HAC combining autologous fat repair of translabyrinthine craniotomy defects and repairing spontaneous cerebrospinal fluid leaks demonstrate efficacious outcomes (2, 3, 5). Especially, when the resection scope of skull osteoma cannot be better evaluated before operation, HAC cranioplasty is adopted according to the actual cranial defects after osteoma resection, so as to avoid the embarrassment of second-stage cranioplasty or the size mismatch between intraoperative resection range and preoperative scheduled repair materials.

In our report, the lack of information on repair materials makes it difficult for us to reasonably interpret the preoperative imaging findings; meanwhile, it also brings a lot of confusion for the preoperative diagnosis. Frankly, the preoperative diagnosis of the osteoma relapse was reluctant. Of course, the patient did receive craniectomy and HAC cranioplasty, which was from the intraoperative confirmation, due to frontal osteoma over a decade ago although the related data of the first surgery were lost. The intraoperative findings included the three-sided fixation of HAC and multiple lesions on the HAC without any attachment to the surrounding normal skull and the roots of lesions originating from the intact dura matter surface through several reserved holes. Honestly, these intraoperative findings were beyond what we thought the surgery would be. Multiple osteoma relapse without any attachment to the skull after HAC cranioplasty is extremely rare, which is firstly reported in the literature to our knowledge. In our case, the patient was highly suspected of relapsing on the third year after surgery according to his medical history description, but the patient chose conservative treatment without any discomfort. In the subsequent time period, the slow growth of osteomas with mild pathological characteristics might better explain why the relapse time was so long in this patient. More importantly, how to explain the pathogenesis more reasonably?

In fact, the ossification of the roots on the intact dura surface was also suspected during operation, which was excluded by pathology results ultimately. Given the presence of multiple cranial osteomas, the patient underwent a colonoscopy, which revealed a negative result, to rule out Gardner syndrome (6). Although the related data of the first operation was lost, and what happened during the first operation was unknown, a logical speculation that the osteoblasts of osteomas from the previous operation would implant in the dura matter may state the pathogenesis; however, it is very difficult to be confirmed. If that happens, intraoperative irrigation of residual bone meal is particularly important to avoid recurrence resulting from the osteoblasts of osteomas implantation.

Besides, intracranial subdural osteomas without the involvement of bony structure are rarely reported, with only approximately 20 cases having been reported to date, and the mechanism behind their genesis remains unclear (1, 7, 8). However, considering the similarity between our case and subdural osteomas, both of which are far away from the bony structure, we can draw on the hypothesis, that is, some authors speculating that primitive mesenchymal cells from connective tissue might migrate into the subarachnoid space along the intracerebral blood vessels. Likewise, the osteomas could arise from the dura, since the meninges, comprising pluripotent cells, may function as periosteum (1, 7–10). Although the osteomas are attached to the outer layer of the dura, no dural cells are observed in the pathological examination of these tumors. Considering the rarity of this case, it is indeed difficult to find more relevant literatures to support its pathogenesis. Even so, the pathogenesis of our case is prone to the osteoblasts of osteoma implantation during the first surgery. Therefore, considering the possibility of recurrence, postoperative management is crucial and the surveillance CT scans of each year after surgery should be recommended.

The case leaves us with many doubts, including whether the HAC holes are just a coincidence to promote their growth and whether the osteomas will relapse again during the long follow-up because there was no special treatment, just cauterization, for the intact dura at the second surgery.

## 4 Conclusion

In this case report, we present an extremely rare and previously unreported case of skull osteoma relapse without any attachment to the skull after HAC cranioplasty. The pathogenesis of osteomas without bone involvement is prone to the osteoblasts of osteoma implantation during the first surgery.

## Data availability statement

The raw data supporting the conclusions of this article will be made available by the authors, without undue reservation.

## Ethics statement

The studies involving human participants were reviewed and approved by the Ethics Review Board of the West China Hospital of Sichuan University. The patients/participants provided their written informed consent to participate in this study. Written informed consent was obtained from the patient for the publication of this case report.

## Author contributions

All authors contributed to the diagnosis and treatment of the patient. XY performed image data analyses and wrote the manuscript. YL contributed to the operation and prepared the manuscript. YZ helped perform the analysis through constructive discussion. All authors have contributed to the manuscript and approved the submitted version.
